# Intestinal Perforations from within by Foreign Bodies*Read at the meeting of the South Texas Medical Association, at Cuero, Texas, June 14, 1899.

**Published:** 1899-07

**Authors:** B. E. Hadra

**Affiliations:** San Antonio, Texas


### TEXAS MEDICAL JOURNAL.

ESTABLISHED JULY, 1885.

PUBLISHED MONTHLY.--SUBSCRIPTION $1.00 A YEAR.

VOL. XV. AUSTIN, JULY, 1899. No. 1.

### Original Contributions.

For Texas Medical Journal.

### Intestinal Perforations From Within by Foreign Bodies.*

BY B. E. HADRA, M. D., SAN ANTONIO, TEXAS.

Perforations of bowels from within by foreign bodies occur but rarely, judging merely from the scantiness of literature. Perhaps, though, they are greatly overlooked, as they mostly create little or no trouble, or if so, because they are rarely taken into consideration. I am sure that if such accidents were more systematically included in the list of possibilities in differential diagnosis, a remarkable frequency of them would be the result. It is the aim of my short paper to say a few words, and to relate a few cases, in support of my assertion.

The foreign bodies will, as a matter of course, find their entrance principally through the natural alimentary doors, and it will, as a rule, take a long time before the consequences will call for medical or surgical interference. By that time, the connection between accident and consequences, if it ever was guessed, will be lost. Thus, for instance, a spicula of bone may have been swallowed, and only after weeks and months the havoc may appear in some obscure form, baffling all diagnostic ingenuity. Perfectly smooth and fine bodies,

*Read at the meeting of the South Texas Medical Association, at Cuero, Texas, June 14, 1899.

like needles, are known to travel through the whole body without doing much harm, whilst more massive or rough objects will be caught in their attempt to break through the bowel wall, and will have to slowly work through by ulceration. They will hereby irritate the continuous or contiguous tissues, produce an adhesive inflammation, and by progressing in their travel get out of their original location, either allowing the hole in the bowel wall to close behind them, or leaving a permanent opening through which bowel contents will escape, producing then an abscess, which may persist indefinitely if it has become well walled off from the general abdominal cavity. The body itself may remain in the abscess, or may escape through the bowel or come to light after the most venturous travel.

Let me now relate two cases which, though the foreign bodies were not found, are easiest explained by such occurrences.

An elderly lady was suffering for many years from a kind of irregular bowel disorder, coupled with pains in the right abdominal side. She never had any female trouble, neither had she had any acute bowel disease. Only occasionally she would feel a little feverish towards evening. Examination revealed a hard mass to the right, and about two inches below the navel. It could also be reached from the vagina when pressed down. It was in no connection with any pelvic or abdominal organ. Operation disclosed the existence of a mass consisting of two shanks of a thickened loop of small intestine. When they were detached from each other a perforation was easily seen in each, and opposite each other, obviously having been tightly adherent. The piece of intestine containing the two holes was resected, and she made a slow but perfect recovery.

In the absence of any other explanation, I deem it a case of perforation by a foreign body, which had penetrated the intestinal wall, had caused adhesive peritonitis with the contiguous wall and finally perforated this also, leaving a permanent fistula between the two pieces of intestine, with irritative thickening of the surrounding tissues.

A very similar case was that in a young Italian woman who had been subjected to very vigorous uterine treatment for a supposed endometritis and oophoritis. The parts were so much inflamed that I, too, was misled, and performed oophorectomy on the right side, where the pains were most severe, and where a swollen ovary was found. Not improving and the signs of a chronic appendicitis becoming more outspoken, she was anew subjected to a laparotomy. Now, an adhesion of the coecum to a loop of the small intestine, about two inches apart from the appendix, was found. A kind of an abscess cavity had formed, walled in by the adjoining intestines. When freed, then a perforation in the coecum and one in the small intestines came to light. They were both stitched up, the cavity well cleaned, and to be on the safe side, the appendix was removed. She slowly regained her health. I think there was about the same condition as in the first case.

One can hardly help thinking, in this connection, of appendical perforations, and, in fact, they are incidents of the same nature. Hard fæcal masses represent here mostly the foreign bodies, and all possible consequences will be met with, either a free perforation into the abdominal cavity or a penning off by omental or intestinal adhesion, or absorption, and so on. A strange case of appendical perforation seems to me worthy of description. I saw it in Dr. Wilkinson's practice in Galveston. A Catholic sister was operated on by him for an enormous accumulation of matter within the abdominal parietes, say between muscular layer and deep fascia. Several gallons of pus were evacuated. As discharging continued out of the incision wound, a second search was made, and a communication with an intraperitoneal sack was detected, which turned out to be a cavity formed by thickened fibrous tissue belonging to the transverse fascia around the sloughed end of a much distended appendix. The location of the opening was above the umbilicus. I think a simple inflamed appendix would hardly have led to a perforation of the transverse fascia—only the active help of a foreign body explains such an occurrence.

However, I feel very well that my position, being based only on supposition and speculation, is not as strong as you may wish. It therefore gives me the greatest satisfaction to produce to you a veritable corpus delicti of such a perforating foreign body in the extraordinary specimen before me. The history of the case is very simple. An Italian of 65 years of age was brought to the city hospital of San Antonio with an incarceration of an old right inguinal hernia after futile attempts at reduction, lasting four days. The contents of the hernia were evidently omental, but there was also a very hard tumor in it of about the size of a hen's egg, which I could not interpret. The operation consisted of tying off an omental mass containing that tumor, and, of course, in adding the closure of the canal. That tumor you see here before you. It consists of firm fibrous tissue, covered with omental fringes, and after dividing it a slim bone—I presume from a fowl—about two inches long presented itself imbedded in that tumor. There is evidently no other explanation but that the bone, after perforating the intestinal wall, became surrounded by omentum, and that the long lasting irritation had led to the thickening of the latter.

But let me now add a few words as to intestinal perforation by foreign bodies in another locality, because it is of still more practical importance. I mean the perforations occurring in the rectum. These are not at all rare, and you are certainly fully acquainted with them.

But let me call your attention to one form which is sometimes somewhat obscure in its features. It is the perforation of the posterior rectal wall above the so-called pelvic diaphragm, say above the line of the coccyx. A body gotten in there has little chance to free itself. It is too difficult to get out downwards through the muscles and fasciæ, and from getting back into the rectum it is mostly prevented by the formation of a pocket in which it is caught. Thus these cases are getting mostly very formidable and long-lasting. Some in my practive have presented themselves as chronic periproctitis, inflamed hæmorrhoids, rectal ulcers, etc., and only late the right diagnosis was made. It was then even necessary to lift the posterior rectal wall in a number of cases, and to cut through the diaphragm, and also to remove the decayed coccyx. I not always succeeded in finding the foreign body. One case, though, which I attended to last year was crowned with success at once. It was a patient who came from a California hospital to ours in San Antonio. He had been operated on several times for periproctitis and other troubles. Still, he was no better. The anus was surrounded by stiff inflamed tissues, and they reached up several inches in the rectum. Under chloroform, a fistula was detected in the posterior rectal wall about two inches high. This was freely incised downwards, and with a great amount of debris, a fish scale came away. He then slowly recovered.

Cases where foreign bodies become partially imbedded in the rectal walls are no doubt sufficiently familiar to you.

---

**The gifted** and versatile Dr. I. N. Love was elected President of the Association of American Medical Editors at the meeting of the Association at Columbus, Ohio, in June.

